# Primary branchiogenic carcinoma: malignant degeneration of a branchial cyst, a case report

**DOI:** 10.1002/cnr2.1315

**Published:** 2020-12-09

**Authors:** Giuseppe Colella, Ciro Emiliano Boschetti, Chiara Spuntarelli, Davide De Cicco, Immacolata Cozzolino, Marco Montella, Gianpaolo Tartaro

**Affiliations:** ^1^ Department of Multidisciplinary Medical Surgical and Dental Specialties, University of Campania “Luigi Vanvitelli” Naples Italy; ^2^ Department of Neurosciences, Reproductive and Odontostomatological Sciences University of Naples “Federico II” Naples Italy; ^3^ Department of Mental and Physical Health and Preventive Medicine University of Campania “Luigi Vanvitelli” Naples Italy

**Keywords:** branchial cyst, head and neck, PBC, primary branchiogenic carcinoma, surgery

## Abstract

**Background:**

Primary branchiogenic carcinoma (PBC) is an extremely rare and poorly documented disease developed from a brachial cleft cyst.

**Case:**

A 51‐year‐old patient was referred to our unit for an upper neck mass. PBC was confirmed in accordance with Kahfif's diagnostic criteria. Prophylactic selective neck dissection was performed in a second‐stage surgery to ensure the complete removal of the neoplasm. Branchiogenic origin with lymphoid tissue was confirmed in the “host cyst” after histological examination and no other tumors were found elsewhere. Regular follow up documented no relapse 12 months after surgery.

**Conclusions:**

Although rare, PBC must be suspected in presence of cervical masses, especially in patients older than 40 years. A standardized treatment algorithm still lacks, but prophylactic selective neck dissection could be considered as the first line choice after the diagnosis has been confirmed.

## 1 INTRODUCTION

Firstly described by Volkmann in 1882,^1^ primary branchiogenic carcinoma (PBC) is a squamous cell carcinoma arising in a branchial cleft cyst. It is an extremely rare, poorly documented, and highly controversial entity,^2^ whose nature remained unclear for a long time.^3^ Recognizing a PBC still represents a challenge for both clinicians and pathologists, because of numerous differential diagnoses encountered, as well as the cystic lymph node metastasis (associated with a primary squamous cell carcinoma localized in the tonsils, in the base of the tongue, in the nasopharynx, in the larynx or in the hypopharynx) and the papillary thyroid carcinoma.^4^

In 1950, Martin et al.^3^ reported a review of the then published literature of possible PBC cases, by which he proposed for the first time a 4‐point diagnosis algorithm, based on anatomical, prognostic, and histological criteria. This first version was then revised by Khafif et al.^5^ in 1989, who pointed the attention on the presence of transition from a normal to a malignant epithelium, with premalignant epithelial changes within the branchial cleft cyst, defining an important criterion to confirm the diagnosis.

Although the defined diagnosis criteria are currently in widespread use, PBC still give rise to some controversies on its nature and treatment choice,^6^, ^7^ due to the unclear prevalence and insufficient epidemiological and prognostic data. We present a case of a middle‐age man affected by a lateral neck mass diagnosed for a PBC treated in our Unit by selective neck dissection alone followed strict follow‐up.

## 2 CASE REPORT

**FIGURE 1:**
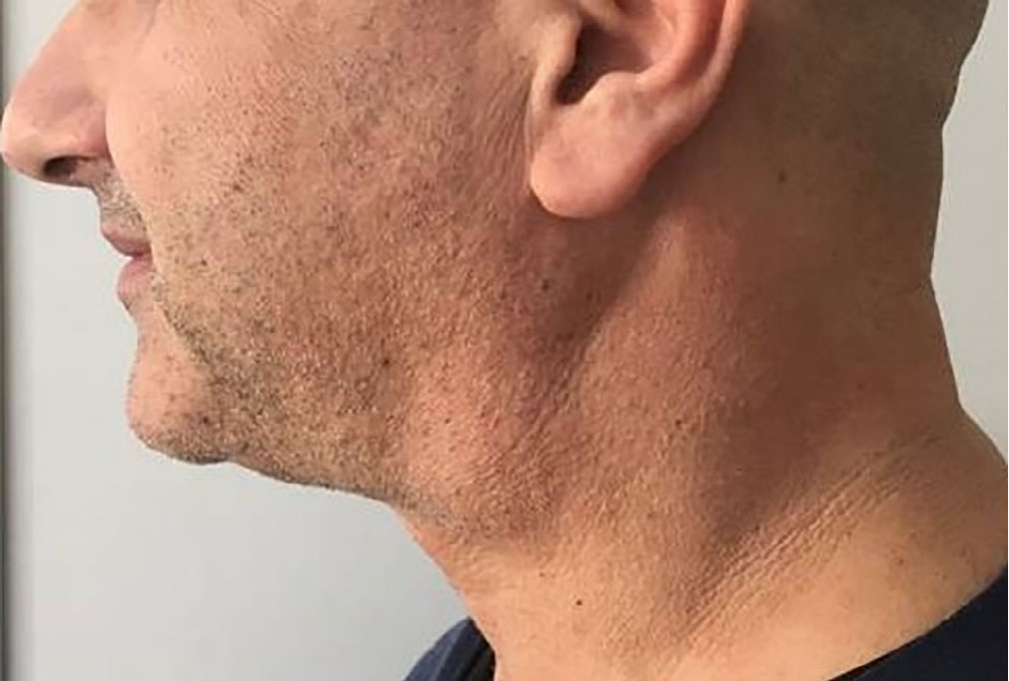
Clinical examination showed a 50 mm, defined, non‐mobile, and painless mass located anteriorly to the sternomastoid muscle

**FIGURE 2:**
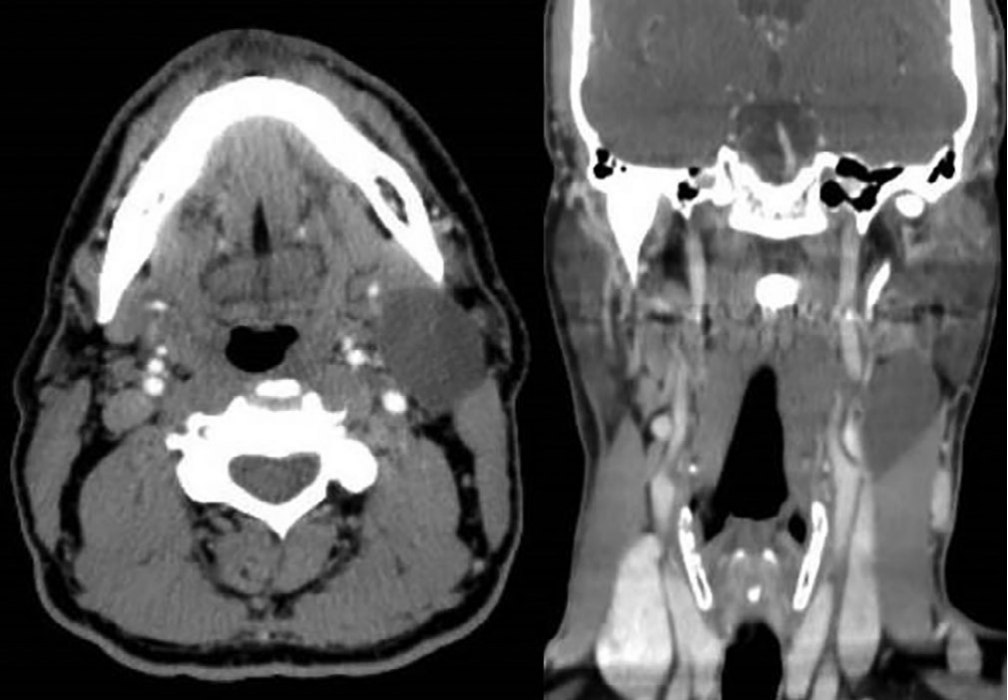
Computer tomography showed a hypodense, homogenous cyst located lateral to the great vessels, anterior to the sternomastoid muscle, and posterior to the submandibular gland

A 51‐year‐old male patient, non‐smoker, was referred to our Maxillofacial Surgery Unit (University of Campania “Luigi Vanvitelli,” Naples, Italy) with a left upper cervical mass of 1‐year duration. Clinical examination revealed a fluctuant, painless mass close to the middle third to anterior border of sternocleidomastoid. The overlying skin appeared normal (Figure 1). Neck ultrasonography showed a well‐defined cystic mass with thin hyperechoic wall and internal anechoic component, measuring 30 × 35 mm. There were no loco‐regional lymphadenopathies. Contrast‐enhanced computed tomography (CT) of the neck showed a circumscribed cystic mass of 30 × 30 × 44 mm surrounded by peripheral enhance, located behind the left sub‐mandibular gland, lateral to the left great vessels, and deep to the left sternocleidomastoid muscle (Figure 2). The patient had previously performed two consecutive FNAC in two different hospitals. By the first FNAC, straw‐yellow material was taken which microscopically showed histiocytes and crystals. The second FNAC showed amorphous material, crystals, inflammatory elements, histiocytes, and anucleate keratin lamellae in a serous background. These features, associated with the clinical‐instrumental presentation, suggested a branchial cleft cyst.

**FIGURE 3:**
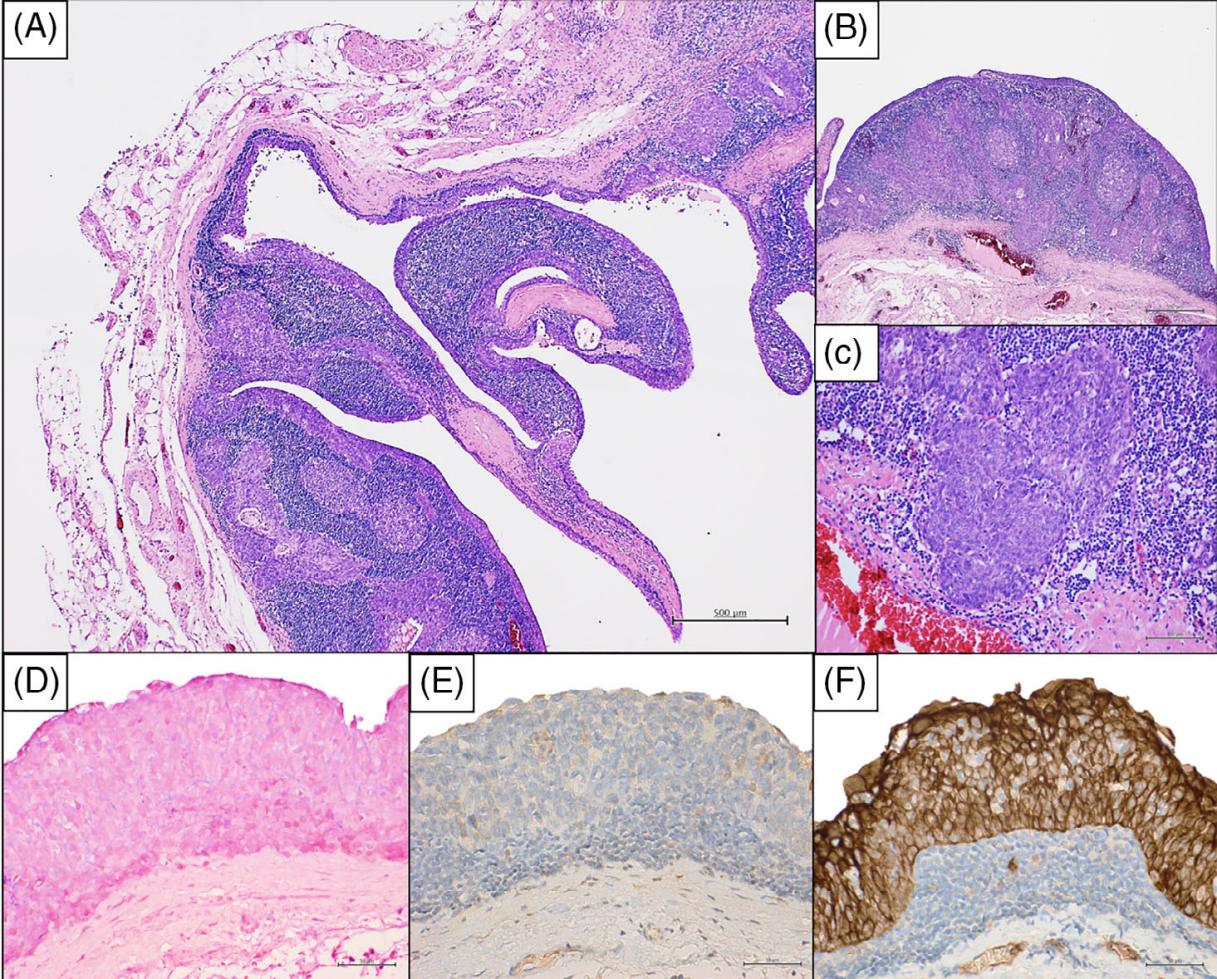
(A) Microscopic observation showed a cystic structure lined with benign squamous epithelium progressing to carcinoma in situ and frank poorly differentiate carcinoma (H&E stain, 2X, scale bar: 500 μm). (B) Infiltrating poorly differentiated squamous cell carcinoma surrounded by lymphoid tissue (H&E stain, 10X, scale bar: 500 μm). (C) A detail of the infiltrating area into the stroma (H&E stain, 20X, scale bar: 100 μm). (D) EBV analysis via in situ hybridization for EBER was negative (ISH, 40X, scale bar: 50 μm). (E and F) Immunohistochemical analysis was negative for p16 (E) and positive for CKAE1/AE3 (F) (ABC, 40X, scale bar: 50 μm)

The patient underwent neck dissection to remove the cyst, which appeared encapsulated. Grossly, the cystic structure measured 3.5 × 3.2 × 2.7 cm and showed a brownish, smooth external surface. Hematoxylin and eosin‐stained sections showed a cystic lumen surrounded by squamous cell epithelium. The epithelium presented a progression from benign squamous epithelium to carcinoma in situ, up to a squamous carcinoma infiltrating the cystic wall even with poorly differentiated areas (Figure 3). Organized lymphoid tissue with lymphoid follicles was present in the cyst wall. These findings identified a squamous cell carcinoma with a gradual transition from benign epithelium to squamous cell carcinoma arose in a branchial cleft cyst. The immunohistochemistry showed negativity for HPV and EBV and positive for CKAE1/AE3. The patient underwent a magnetic resonance (MR) of the head and neck region followed by a 18 FDG PET‐CT scan and a fiberoptic nasopharyngoscopy and laryngoscopy that revealed no lesions or abnormalities, defining any occult primary tumor, thus diagnosis of PBC was confirmed. The case was discussed in the multidisciplinary team meeting (MDT) and it was established to perform prophylactic selective neck dissection to ensure the complete removal of the neoplasm. Postoperative period passed without complications. All lymph nodes resected were negative at the histological examination. The MDT recommended a follow up at closer intervals during the first year, adjuvant therapy was not suggested. Neck ultrasonography was performed 3, 6, and 12 months after surgery and no signs of relapse were reported.

## 3 DISCUSSION

PBC is described as malignant transformation of the normal lining epithelium of the cyst. Chronic inflammation and irradiation may cause or promote this malignant degeneration,^8^ although its etiopathogenesis remains unclear. Differential diagnosis between a primary carcinoma of a branchial cyst and cystic metastasis of the neck still represents a challenge nowadays. According to Martin et al.,^3^ diagnosis of PBC can be confirmed if each of the following criteria are respected: (a) location of the tumor along a line anterior to the sternocleidomastoid muscle between the tragus and the clavicle; (b) histologic appearance of the tumor is consistent with tissue present in the branchial vestige; (c) the clinical course of the disease is that no primary tumor occurs within a 5‐year follow‐up period after diagnosis; (d) histologic evidence of a cancer developing in the wall of an epithelial‐lined cyst situated in the lateral aspect of the neck. Khafiff et al.,^5^ in 1988, revised these criteria in order to focus on the histological aspect, in particular on the presence of gradual transition of normal squamous epithelium to carcinoma. According to Khafiff, diagnosis of PBC is confirmed if: (a) the tumor is located in the anatomic region of the branchial cleft cyst or sinus as defined by Martin et al.; (b) the histologic appearance of the tumor is consistent with its origin from branchial vestiges, that is, squamous cell carcinoma; (c) the presence of carcinoma within the lining of an identifiable epithelial cyst is confirmed; (d) the transition from normal squamous epithelium of the cyst to carcinoma is identified; (e) the comprehensive evaluation of the patient did not report any other primary malignant tumor. All five points were respected in the presented case.

In order to exclude any primary tumor are recommended a meticulous clinical evaluation of the oral cavity, cervical lymph nodes, and thyroid gland (along with hormonal profile) followed by fibroscopy of the upper aerodigestive tract, magnetic resonance, and PET/CT total body. Some authors suggested to perform bilateral tonsillectomy, Waldeyer's ring biopsy, or random pharyngeal biopsies in patients positive for HPV and EBV, even in absence of macroscopically suspicious lesions resulting from instrumental evaluation.^6^, ^7^, ^9^ Since our patient was negative to HPV and EBV and no oro‐rino‐pharyngeal lesions were detected, these procedures were not performed in accordance to other Authors' opinion.^10^, ^11^, ^12^

Yehuda et al.^13^ found that 3‐24% of lateral cystic masses of the neck region are associated with malignant neoplasms, but this percentage increase up to 80% in patients older than 40 years. Most of them are represented by metastatic lymphadenopathies of primary tumors of other head and neck region^4^, ^13^ but PBC should be suspected until proven otherwise. Early diagnosis is mandatory to prevent the progression of the disease, but the treatment of choice is not unanimous between Authors, since large differences can be found, probably due to the leak of significative data regarding the prognosis. By the way, some authors suggested only to follow up, others recommended postoperative adjuvant radiotherapy or chemoradiotherapy or modified‐radical neck dissection followed or not by adjuvant radiotherapy.^14^, ^15^, ^16^, ^17^ Our choice was to perform a selective lymph nodes neck dissection, according with the MDT decision, although areas of poor differentiated carcinoma were described. After surgery, the MDT decided only to follow up the patient without other interventions, due to the absence of positive nodes at the histological examination.

## 4 CONCLUSIONS

Although Kahfif et al. defined well‐established diagnostic criteria, clinical, and surgical management of PBC are still debated between Authors. Since it was firstly described by Von Volkmann in 1882, many algorithms of treatment have been proposed, ranging from conservative strategies to really aggressive ones. We recommend an early surgical treatment of all cystic masses of the neck in patients older than 40 years, eventually followed by a second stage surgery to ensure the complete removal of the neoplasm. Moreover, the existence of PBC should be considered if diagnostic criteria are respected, although its rarity.

### HUMAN AND ANIMAL RIGHTS

No animals were used in this research. All research procedures were in accordance with the Helsinki Declaration of 1975, as revised in 2008.

## CONSENT FOR PUBLICATION

The study has been approved by Institutional Ethical Committee. Informed consent for publication was obtained from the patient.

## CONFLICT OF INTEREST

The authors have stated explicitly that there are no conflicts of interest, financial supports, or otherwise in connection with this article.

## AUTHOR CONTRIBUTIONS

**GC:** Investigation; project administration; supervision; validation. **CEB:** Data curation; investigation; methodology; writing‐original draft. **CS:** Data curation; investigation; methodology; writing‐original draft. **DD:** Conceptualization; formal analysis; visualization; writing‐review and editing. **IC:** Data curation; investigation; methodology; writing‐original draft. **MM:** Data curation; investigation; methodology; writing‐original draft. **GT:** Supervision; validation.

## Data Availability

The Authors allow the Journal to share all the published data, although there is not an online repository which they have been collected in
